# Intensive Care Managers' Experiences of the COVID-19 Pandemic: A Dramatic Change of the Intensive Care Landscape

**DOI:** 10.1155/2023/3052994

**Published:** 2023-09-29

**Authors:** Anna Nordin, Åsa Engström, Maria Andersson, Angelica Fredholm

**Affiliations:** ^1^Karlstad University, Department of Health Science, Faculty of Health, Science and Technology, Karlstad, Sweden; ^2^Lulea University of Technology, Department of Health, Education and Technology, Division of Nursing and Medical Technology, SE-97187 Luleå, Sweden; ^3^Swedish Red Cross University, SE-141 21 Huddinge, Sweden

## Abstract

**Aim:**

To describe intensive care managers' experiences of premises and resources of care in intensive care units during the COVID-19 pandemic.

**Background:**

Intensive care units (ICUs) were enormously pressured during the COVID-19 pandemic from many ill patients, requiring advanced care. Hospital and community volunteers increased staff strength. Obligatorily, recruitments were also conducted using transfer of staff from different hospital departments. However, there is little knowledge about intensive care managers' (ICMs) experiences of leadership during the COVID-19 pandemic.

**Methods:**

A qualitative descriptive study was conducted from March to April 2022. Semistructured interviews were held with 12 ICMs who were purposively sampled from the ICU in ten Swedish hospitals. Data were analysed using qualitative content analysis.

**Results:**

Two themes emerged: a dramatic change of the intensive care landscape and we could handle more than we thought, but at a steep price. Participants described that the ICUs had to perform extraordinary changes at a very fast pace, which initially created a sense of cohesion. Training and introduction to war-like conditions associated with uncertainty meant that ICMs had to support ICU staff in prioritising interventions. Participants described how ICUs stood strong against a pandemic, but stress, worries, and anxiety took a heavy toll on ICU staff and ICMs. The pandemic eroded the resilience in ICUs. Participants described a deterioration in health and said that sick leaves and resignations occurred.

**Conclusion:**

Our findings show ICMs' experiences as a field of tension between resources and demands, whereby the changes created a heavy burden that left intensive care weakened. *Implications for Nursing Management*. Findings emphasised the importance of creating working conditions using human resources and materials in order to rebuild resilience in intensive care with the ability to conduct safe patient care.

## 1. Introduction

During the COVID-19 pandemic crisis, healthcare systems worldwide were enormously pressured by the millions of people who became ill, requiring advanced care in intensive care units (ICUs) [1–3]. ICUs faced challenges that had rarely been seen, such as increasing the physical and clinical capacity for care of highly contagious patients [4], which created unique demands for intensive care managers (ICMs) [5] in a way most had never experienced.

The clinical team providing care in an ICU is specially qualified, interdisciplinary, and interprofessional [6]. However, to meet demands, caused by the COVID-19 pandemic, healthcare organisations transferred and paired general care nurses and postgraduate nurses from specialties' such as anesthesia, operating theaters, and emergency rooms together with intensive and critical care nurses (ICNs) to increase the numbers of patients that could be treated in ICUs [7–9]. ICMs' coordination of large interdisciplinary teams requires administrative structure with designated medical responsibility, coordination of staffing, care needs, and establishing policies and priorities for ongoing patient care [6]. During the COVID-19 pandemic, the staffing coordination had to be organised in a new way. For example, the nurse-to-patient ratio in Swedish ICUs increased from 1 : 1-2 to 1 : 3 and was sometimes even higher [10]. At the same time, ICMs and healthcare managers feared the safety and risk for infection for their own staff, as well as anxiety and overload in the work situation, and support from higher organisational levels became significant [11].

According to the National Corona Commission [12], Sweden's pandemic preparedness was marked by a slowness of response. The first patient with a COVID-19 diagnosis admitted to a Swedish intensive care unit (ICU) was on March 6, 2020, and during the year, a total of 4222 COVID-19-diagnosed patients were cared for in the ICU [13]. Before the pandemic, ICU capacity in Sweden was around 5 ICU beds per 100,000 inhabitants, one of the Western world's lowest numbers of ICU beds. During the first wave of the pandemic, Sweden doubled its ICU capacity by setting up temporary ICU beds (OECD/European Observatory) [4].

Preparedness, decision-making ability, and actions taken by organisational leadership in crisis situations are essential for managing resources and might affect patient outcomes [14, 15]. Adaptations in ICUs have been achieved at the cost of extreme pressure on staff and cancelled and postponed care [12]. The leadership has a crucial role in crisis situations, and important attributes are clear and fast communication, decision-making and fair prioritisations, building trust, and the leader competence [16].

In summary, the COVID-19 pandemic generated a worldwide sudden change in human health and healthcare, especially in intensive care. While several studies have focused on the critical care nurses' experiences and highlighted the need of good leadership and resilient organisations, there is a lack of studies focusing on the perspective of ICMs' experiences of leadership during the different surges of the COVID-19 pandemic. Therefore, the aim of this study was to describe intensive care managers' experiences of premises and resources of care in intensive care units during the COVID-19 pandemic.

## 2. Methods and Design

The conducted research is methodologically positioned within the interpretative qualitative research paradigm. The phenomena under study were explored and interpreted inductively in order to capture ICMs' first-hand experiences.

The inductive, qualitative study design included semistructured interviews that were conducted using an interview guide. The design provided the opportunity to access the ICMs' experiences. The data collected were thereafter analysed through qualitative content analysis [17]. The study method was compliant with the Consolidated Criteria for Reporting Qualitative Research (COREQ) checklist [18].

### 2.1. Setting

Using the Swedish Intensive Care Registry, Swedish hospitals whose ICUs had at least 40 COVID-19 care sessions in the middle of 2020 were located. Prior to the pandemic, the ICUs in Sweden had one of the lowest capacities in the EU. Due to the high number of COVID-19 patients, the Swedish ICUs were at 80% capacity, a percentage that could have been even higher if there had been more ICU specialist staff available [4]. In addition, there was initially no medical treatment or vaccine against the disease in Sweden or in all of Europe [19]. A shortage of qualified staff meant that anyone with an interest and opportunity could sign up voluntarily for work in ICUs, particularly in the big city areas. The ICUs faced shortages of personal protective equipment (PPE) for ICU staff, and some hospitals were forced to recycle disposable PPE after sanitation [4].

### 2.2. Participants

Executive managers in intensive care departments in hospitals in the middle and north of Sweden were asked to give their consent to allow their organisations to participate in a study aimed towards ICMs experiences during the COVID-19 pandemic. Twelve managers with strategic and operative responsibility for ICUs at ten hospitals participated in the study. The participant group consisted of those who had worked as active ICMs during the COVID-19 pandemic. Among them were first-line managers with an operative role; others had strategic roles, such as hospital managers and managers for specific professional groups. Both male and female ICMs participated. All managers had an active background as a specialist physician or specialist nurse in intensive care, and all were cohabitating or married with children and between 40 and 64 years old. All managers had had the experience of introducing new intensive care staff, including anesthesiologists as colleagues and postgraduate nurses as managers.

### 2.3. Data Collection

Thirteen ICMs at ten hospitals were identified via purposive sampling and approached via e-mail to participate in the study. One ICM who was approached did not respond to the e-mail invitation to the study. Data were collected remotely between March and April 2022 via digitally recorded interviews. This enabled compliance with Swedish social distancing requirements [20], while data collection from geographically distant participants could be arranged. The ICMs (Table 1) chose the time and location for the interview. Seven preferred the workplace and five chose their homes for their interview. The interviews were conducted separately by two of the authors (AN, *n* = 6 and AF, *n* = 6).

Data were collected through individual semistructured interviews, which lasted from 45 to 65 minutes, and these were voice-recorded and transcribed verbatim. Field notes were used and considered during the interpretation of each interview as a whole. The interview guide was constructed with open-ended questions to provide an opportunity for the ICMs to describe their own experiences (Table 2). The questions revolved around their management experience in intensive care units during the COVID-19 pandemic with special focus on critical care organisation in crisis, introduction of ICU staff with no previous experience in healthcare, as well as the introduction of ICU staff with experience in healthcare in general and intensive care in particular. Questions also revolved around priorities during the pandemic, supervision of students during a chaotic phase in healthcare, and their personal experience of organisational support. Follow-up questions were asked to deepen the dialogue and enable reflection.

### 2.4. Data Analysis

The authors applied qualitative content analysis, as described by Graneheim and Lundman [17], to the interview text. Each interview was read through several times in order to gain a sense of the content as a whole. The entire text was then read in order to identify meaning units, guided by the aim of the study. The meaning units were condensed and sorted into subthemes related by content, constituting an expression of the manifest content of the text. The subthemes were related to each other and were then subsumed into two themes, i.e., threads of meaning that emerged in the categories. The authors checked the analysis independently and then discussed their findings before reaching a final agreement.

### 2.5. Ethical Considerations

The study was conducted according to the Code of Ethics of the Declaration of Helsinki and adhered to the principles of confidentiality, integrity, right to self-determination, and privacy, as well as transparency and secure data processing. The study was given ethical approval by the Swedish Ethical Review Authority (Dnr 2020-04428). Along with a request for participation in the study, the participants received an information letter describing the aim and context of the study, principles for confidentiality, and the right to abort the study at any given moment. The researchers who conducted the interviews were not involved in any work at any of the ICUs where the ICMs were employed.

## 3. Results

**Figure 1:**
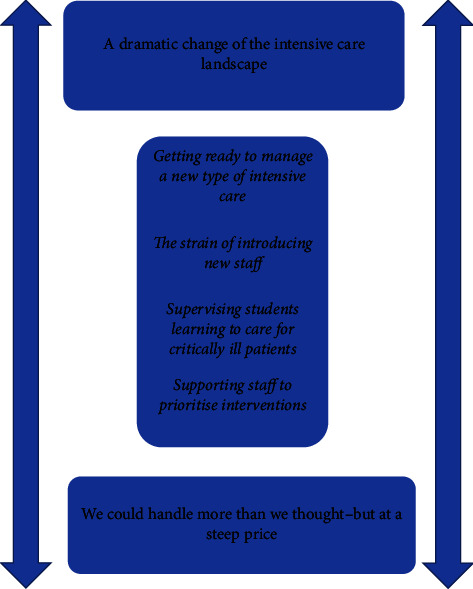
Findings on how the subthemes relate to the field of tension.

The results showed that there is a dynamic force between a dramatic change of the landscape in intensive care and a personal and organisational cost that the change entailed. The result is structured as a field of tension with the two themes: a dramatic change of the intensive care landscape and we could handle more than we thought, but at a steep price, and their respective subthemes. Both the themes and the subthemes are two-sided, that is to say, all show some of the dramatic change in the intensive care landscape as well as the significant cost of these results (Figure 1). What is consistently reflected is not only the ICMs' experiences and perceptions but also their narratives and interpretations regarding what happened in the organisation, within staff groups, and to themselves personally. Each of the subthemes within its overarching theme is described with examples of quotes from the participants' transcripts. In order to not reveal the 12 participants, none of the reported quotes include demographic descriptors, such as the age or unit assignment of the participants.

## 4. A Dramatic Change of the Intensive Care Landscape

The COVID-19 pandemic constituted an unprecedented change in intensive care for ICMs and ICU staff. This change was distinguished by how quickly everything had to be done. Initially, the scale-up was characterised by a sense of cohesion and job satisfaction that stemmed from the fact that everyone understood that now was the time to stick together and take action. The somewhat war-like state could also, according to ICMs, trigger those trained in emergency medical care and intensive care to be allowed to use their knowledge. In this theme, a very large organisational change and its accompanying problems with prioritisation of resources are depicted.

### 4.1. Getting Ready to Manage a New Type of Intensive Care

The ICMs indicated that in order to manage intensive care with a sharply increasing number of patients, they were forced to quickly rearrange the premises and installations, since the modern ICUs with single rooms were too demanding of staff. Although the work had to be carried out under great time pressure amid much uncertainty, the ICMs describe that they as well as the ICU staff felt that the healthcare system was put to the test.

Because it was, I mean, both the situation with the intensive care was completely different from any other intensive care we have ever conducted…and then you were worried about yourself. You were worried about your family at home. How would we keep from infecting each other? We would not infect each other, but there were—there were so many levels of that worry. ICM 1.

ICMs depicted how patients and ICU staff had to be moved to large open areas and open care floors such as postoperative units and recovery units where ICU staff could monitor several patients simultaneously, which meant that ICNs could move rather unhindered between different patients and communication was easy.

ICMs reported that the acquisition of medical equipment occupied a large part of the work. When the number of patients in need of critical care exceeded the ICU's capacity, managers described how they had to use anesthesia devices, old ventilators, and materials from storage. As the disease was new and unknown, the ICMs described a fear that the ICU staff would get sick and possibly die. Availability of, trust in, and use of personal protective equipment (PPE) occupied a lot of ICMs' work. Communication with patients, relatives, and colleagues was affected by the PPE despite large name tags, and the staff had to raise their voices and even shout, making it very hard to have personal dialogue and confidential conversations even though they could not arrange it in a better way.

But you can understand this afterwards. Of course, you're used to being able to sit quite close and have a dialogue with people and stuff like that; suddenly, it was impossible. ICM 2.

Since the ICU staff's fear, according to ICMs, partly evolved from being able to protect themselves from infected patients and colleagues, information to ICU staff and lot of cooperation with the hospital's own unit for healthcare hygiene was necessary. The educational challenges meant that ICMs had to inform the ICU staff and to create acceptance and trust for PPE. The ICU staff became worried when instructions and practical performance were contradictory, making them often refrain from battles when ICU staff used PPE in ways that were not recommended, regardless of whether involved in overuse or underuse of PPE. ICMs articulated that they had to choose their battles.

I can still see that, because they discovered that if you wore a cap of some kind, the visor fit better. Then I thought, “I'll take on the necessary battles. That wasn't essential,” period. ICM 3.

ICMs expressed that due to a lack of sufficient equipment and PPE at the hospitals, they had to introduce restrictions in use. The restrictions were difficult to implement since terrified staff took more equipment than necessary. In order not to waste equipment, ICMs had to instruct ICU staff not to go to the bathroom and almost force ICU staff to stay in the patients' rooms so as not to have unnecessary changes. Some ICUs had equipment for only single upcoming shifts, and ICMs describe how they calculated that PPE would last for only four more hours.

And it was hard to disguise so that the staff would not notice it, this with, well, partly the way they were sent in and partly the fact that we didn't have protective equipment. When they asked, I said, “Yes, it's no problem,” but inside my stomach was a big pit. ICM 5.

The work of informing the staff was demanding for the ICMs, as there was constantly new information about the virus, treatments, and protective equipment. They provided a lot of information to ICU staff during the first wave, both in writing and verbally, and had to be very consistent in their information. They explained that there would be changes and there would be new procedures. At the same time, they had to be clear about the importance of following guidelines.

### 4.2. The Strain of Introducing New Staff

When the healthcare system had to mobilise to face the pandemic, ICMs started to recruit ICU staff. The ICMs, whose managers had contacts at European and Chinese hospitals, realised the seriousness of the situation and understood that a quick and powerful mobilisation was required. This clear set of demands was described as somewhat shocking, but with gratitude in hindsight, one ICM stated

I'm very happy that my boss was so determined, and “Now we have to.” Because man, I can say that I myself was like this. Is it so urgent really? Will there be this many patients? ICM 6.

Advertising on social media sites such as Facebook was also used for recruitment. In order to match the need for ICU staff to monitor patients, anybody who applied and wanted to offer service was accepted. It was enough to have someone who could monitor the patients, ICMs said. The broad recruitment resulted in civilians with background from restaurants, theaters, shops, entertainment, and from the military, some ICMs reported. ICU staff close to patients, such as assistant nurses and nurses, were recruited from other units in the region, and ICMs indicated that they had to retain staff with adequate training but who had chosen other jobs because of staffing needs.

According to ICMs, internal recruitment of graduate nurses with an interest in critical care took place, and postgraduate nurses with experience in critical care in a broad sense were recruited internally and more or less forcibly relocated to the ICUs, causing some to be horrified and cry. It was common for nurses from emergency care, anesthesia, and operation rooms to be relocated to the ICUs. Postgraduate nurses in anesthesia (PGA), who were familiar with ventilators, were deemed suitable, as they were confident in handling patients in need of intubation, and thus, they were prioritised to perform those important interventions. Difficulties later arose when the patients were in need of intensive care nursing skills, since PGA skills primarily evolved around short-term care in anesthesia. ICMs reasoned that this was understandable since PGAs had chosen to not work in ICU. There were also ICU staff from other departments who did a fine job.

Then we also had…we also actually hired the new staff. Some came from the other hospital units and then thought that it was so nice here and it was so very good (laughs). So, we actually hired them…at ICU, yes. So, some… Absolutely, there were several who were unbelievably sort of… very able… They, got in this quickly and yeah, they adapted very well. ICM 7.

Experienced and retired ICNs or those who had left intensive care for other types of care were real assets, since they were able to move into the job quickly. ICMs said that it was obvious that special qualities were required from nurses to work in the ICUs. Some were unsuitable and did not cope with the tasks despite a long introduction. They were sorted out because the regular staff discovered shortcomings.

One realised quite quickly that it wasn't really possible to come in as…“nurse anyone” and manage this. It required some kind of…yes…knowledge in the area. ICM 2.

In order to prepare new ICU staff for the assignment, the ICMs had to arrange introductory training for new staff both centrally within the organisation and in their own critical care department. ICMs assigned student managers or experienced ICNs with pedagogical abilities to run the education. Initially, the introduction consisted of ICU staff just being put in patient rooms and having to stick to working the same shift ICMs indicated, but after a while, they had more time to structure the introduction.

To be able to organise the training of a large number of ICU staff, ICMs said they used both analogous aids, such as manuals and checklists, as well as digital aids, such as interactive digital training, recorded lectures, and films with technical equipment and approaches to various interventions. The ICMs described that the introductory training for unexperienced ICU staff started with limited values of importance and was shown how to monitor ICU patients and how to fill in information on an ICU curve. This was followed by bedside training, such as patient care, patient bedding, and changing the patient's position in bed. Several ICMs pointed out that a commonly occurring scope for training was three days and that graduate nurses received longer introductory training than the nurses with postgraduate education. Graduate nurses were introduced to arterial blood gas sampling and care of indwelling urinary catheters. Introductory programs in medical equipment and overviews of various procedures, such as PDM systems, nursing records, lists for drug prescriptions, and documentation systems, were also common.

We had to begin with that, so they couldn't do anything. Then it was because things calmed down a bit, or we had a bit more time to structure it, so we had small internal trainings. ICM 1.

ICMs described how unexperienced physicians were relocated to the ICU, and in order to learn ventilator treatment, they worked alongside an experienced anesthesiologist or intensive care physician. Due to the burden of care, double staffing was required, whereby physicians who worked in anesthesiology were relocated to intensive care units. Physicians without specialist training in intensive care but with experience in orthopedics and surgery were allowed to make rounds, write referrals, keep daily journal entries, and contact relatives.

### 4.3. Supervising Student Learning to Care for Critically Ill Patients

The ICMs disclosed that during the pandemic, both graduate and postgraduate students were conducting hospital-based education, as well as internships, but to a very small extent compared to before the COVID-19 pandemic. Due to the initial lack of PPE and fear that students would become infected, ICMs had to cancel all student activities. That may have also contributed to their disappearing from internships at the beginning of the pandemic, ICMs reported. When restrictions on PPE were eased and students and internships were allowed, masks affected the supervision.

It was difficult to hear what they said. We had those Sundström masks, so you had to shout through them, and if you turned your head away, you couldn't hear what they were saying. So that only that made the supervision… the situation tough. ICM 1.

The multi-ill and complex patients constituted a patient group that was very suitable, especially for postgraduate nursing students, according to the ICMs, and the complexity and multiorgan failures created opportunities to learn a great deal. The ICMs also stated that they were careful to investigate whether any students did not want to care for these patients, but no students made that demand. Where students were allowed, the ICMs described an eagerness to care for patients and to arrange hospital-based education and internships. The students, together with their supervisors, had to care for patients with COVID-19 because no other patients were in the ICUs, and they had to take on the same responsibilities and carry out the same actions as students did before the pandemic.

As they have been with us during the pandemic and seen so many seriously ill intensive care patients, you can say that they are full-fledged ICU nurses when they, uh, in practical terms, when they leave. They have learned an incredible amount. It has, after all… other staff did also. ICM 5.

### 4.4. Supporting Staff to Prioritise Interventions

The absolute majority of ICMs said that both medical and nursing priorities had to be made, as the volume of patients and their needs were far greater than the availability of beds and staff to perform all the procedures. ICMs described that their managers showed little understanding that the staff was exhausted and that general prioritisation regarding the transition to normal mode was necessary.

The medical priorities concerned when and if to end treatment and ICMs reported discussions about the indication for intubation. Initially, treatment was interrupted because there was a lack of knowledge as to whether the patient would be able to recover at all. Once effective treatment for COVID-19 evolved, there was a need for discussions among ICU staff regarding decisions as to when treatment should be terminated. According to ICMs, ICU staff reported ethical stress because of the patients who had been unsuccessfully treated earlier during the pandemic. They shared a fear that priorities made during the COVID-19 pandemic would seep out into the organisation and become the standard level for tomorrow's staff.

In order to balance ICU staff levels and patient needs for ICU nursing interventions, ICMs said that interventions such as change of body position in bed, bedding, bed washing, oral care, showering, and hair washing as well as mobilisation were not prioritised and at times omitted. Central IV-line bandage changing, cleaning, and changing equipment, such as ventilator hoses and infusion lines, were also thinned out due to the need for prioritisation. The ICUs management of medical equipment and drugs were also affected; monitoring of intravenous infusions and injections was carried out with less accuracy.

We had to give up a lot of replacement routines and hoses would sit longer and so on. ICM 1.

The ICMs explained that lower prioritisation of interventions demanded ICU staff to lower their ambition because it was impossible to provide the same level of care as had previously been given. The ICMs indicated little or no ethical difficulties themselves in this—the important thing was that the patients would survive. Some ICMs tried to clearly communicate their responsibility for the down prioritisations and believed that staff doing their best possible was enough. However, it was challenging to motivate employees to perform care perceived as subpar, and staff needed reassurance that it was okay to prioritise other things; otherwise, conflicts could arise. According to one ICM,

In this type of situation, when it was at its worst, it was about saving lives. And I know it caused ethical stress for both employees and managers, and that's what I said before. Maybe you didn't feel proud in every given situation, but it was actually lifesaving. And, of course, sometimes you felt that you were pushing the staff to the breaking point because you didn't really have a choice. ICM 9

The ICMs prepared lists to make it clear to ICNs that they did not have to provide all services that were typically performed in normal circumstances. ICMs also received help from staff to determine the three absolutely most important measures. In addition to supporting the staff in determining which nursing measures were most important, ICMs created opportunities for debriefings with psychologists, occupational healthcare, and other crisis support. The reflection sessions were described by ICMs as urgent, and staff relayed that they were able to persevere in relation to priorities.

## 5. We Could Handle More than We Thought, but at a Steep Price

No, but I would not ever want to be without it really, but I…but I don't want to be part of it again either. I can say that (laughs). ICM 8

The ICMs depicted the COVID-19 pandemic as a period that encompassed efforts that put intensive care at its peak of both shortcomings and strengths. Intensive care had the power to gather and stand against a pandemic, but the activities came with an economic cost and caused stress, worry, and anxiety among people working in the ICUs. Higher prioritisations and care below the perceived lowest level took a toll on ICNs and sick leaves followed. Confidentiality and patient safety were subjected to severe tests as resources to uphold the same level of care as before were not available. In this theme, lessons and insights from a very stressful period in intensive care and its effects on individuals and the organisation are depicted.

### 5.1. Getting Ready to Manage a New Type of Intensive Care

ICMs described a situation where the first intensive period of upscaling of resources demanded an enormous amount of their capacity and their stress tolerance was put to the test as available PPE was insufficient for critical care in Sweden, both in the amount and to meet the requirement level. ICMs indicated that they worked many hours and that spending time with family, training, and social activities became deprioritised. ICMs also talked about their fear of infecting family and loved ones. They were forced to make decisions loosely due to a lack of resources. The systematic quality of personnel work was deprioritised and their work was instead characterised by “putting out fires.” The experience of persuading ICU staff to prioritise which interventions should be carried out and maintaining care for so many seriously ill and infectious patients was described as extremely difficult.

I think that this [caused]ethical stress from our employees as well. They felt that they could not always do as good a job as they would like to do. This obviously transferred to the management so we had to harbor the ethical stress and then we're also expected to maybe come up with solutions that we, that we didn't have. ICM 9

The lack of resources and the insufficient number of experienced ICNs and ICU staff led to the work being carried out unreflectively and thoughtlessly. ICMs also stated that monitoring of the drug supply could not be carried out with the same accuracy as previously, and the initial lack of drugs with Swedish instructions resulted in medical injuries and impaired patient safety. The initial limitation of the ICU staff's access to PPE was perceived as very burdensome, and it caused strong concern. They were not able to inform the staff, even though the managers understood that secrecy was maintained with the best of intentions.

But I think many of us felt that no matter how much you work, it's not enough. We didn't have enough resources to handle this. Many decisions were made on bad grounds. ICM 2.

To manage the increasing number of patients, open care floors in postoperative units were used, and these meant that confidentiality was breached.

They were, you know…old men with their bums in the air. One of them was alert enough to hear what everyone else was saying and then sort of asked: “Yes, but how is Gunnar?” “Yes, do you know him?” “No, but I heard what you're saying.” Just like that, ethics was “pfft.” Yes, we had to do everything that was in one's power, but premises and shielding were not available. ICM 10.

Emotionally, ICMs expressed a feeling of inadequacy in working with their staff, and some indicated that they distanced and isolated themselves from staff at times in order to cope with the situation. This could manifest itself in their not being able to participate in activities with several people in the unit, or choosing to eat lunch in privacy, or leaving the workplace on breaks just to be left alone. The ICMs also said that the heavy workload led to their being short-tempered with each other and that they were forced to shut down their emotions in order to be able to manage and motivate the staff, and that they sometimes experienced tunnel vision and irritability. When surrounding departments were able to carry out personnel work, ICMs described feelings of jealousy and irritation by the situation because they chose not to come and help.

But maybe I've left (the unit) at certain moments of the day, just left. I've gone with my lunch bag to get away for a while. I've closed the door to my office more. I've been working from home more. ICM 8.

### 5.2. The Strain of Introducing New Staff

According to the ICMs, the ICNs were heavily burdened, partly with the introduction of ICU staff in patient care and partly when their own ambition for good care failed due to staff shortages. Various attempts were made to organise responsibility for the patients, where ICNs would have overall nursing responsibility and work together with experienced ICU assisting nurses and ICU staff to care for from one to six patients. The attempts were described by ICMs as organised chaos and often resulted in the ICN's attention being fragmented. It was apparent to those concerned that introduced ICU staff did not administer care that they felt was sufficient.

And again, I return to the fact that our usual staff of nurses and assistant nurses were very heavily employed, because they were supposed to both manage intensive care in a good way, which is in line with the…the feeling that “I'm doing something good.” And then you have to introduce, and then you still had to face that things did not work, because sometimes they didn't…so, I think that many in that group and also among the physicians felt this. Well, you could call it ethical stress or whatever you want to say. ICM 2.

The leadership and responsibilities were sometimes unclear to the ICMs. The introduced ICU staff were caught in limbo as their ordinary managers were unaware of what they did or how they felt, despite their wish for their managers' commitment. Some ICMs expressed that it was nice to avoid responsibility for certain personnel issues with the ICU staff you did not know. At the first wave of COVID-19, newly introduced staff from other nearby units, such as anesthesia, ER, and surgery, were easy to recruit, but interest dropped sharply over time.

It turned out in retrospect that those who had a manager elsewhere actually had NO manager. We tried to bring them into our groups, but it didn't work. I have to say that it wasn't optimal; no one really had time or could do it. Their managers had no idea what they were doing in our unit, how it was like. I don't think I saw anyone's manager who came and checked: “How are my girls and boys doing? how are they being cared for?” They became ours and afterwards they just returned back. It also meant that almost no one wanted to come back [to the ICU] - no one came back in the other waves either. And when we reflected with them, this is what they experienced as the absolute most burdensome thing, [they] had no one…their managers did not see them when they were struggling the most. ICM 1.

### 5.3. Supervising Students Learning to Care for Critically Ill Patients

The ICMs narrated that there was great fatigue among the ICU staff, which meant that staff were unable to supervise students in the same way as usual. The students were able to experience and learn a lot, but the ICU staff had to forego the calm pedagogical, reflective guidance due to lack of time. Those ICUs that had used peer learning as a method had difficulties maintaining this way of working and supervision became scattered. ICMs expressed that supervising postgraduate students could not be done with the same controlled pedagogy as previously.

What has been difficult with supervising, it was most of all, that everyone was so terribly tired. Maybe they couldn't quite cope in the same way…and…I think we had to give up calm pedagogical, reflective supervising (sigh). ICM 1

### 5.4. Supporting Staff to Prioritise Interventions

The ICMs expressed that due to the strained situation for the staff, the resources only permitted short-term essential nursing and medical interventions; other interventions were deprioritised. Patient safety measures, such as overlapping time for reports and nursing documentation, were deprioritised. Not prioritising routines for safe care, lowering the level of nursing, and deprioritising patient-related nursing interventions were major challenges for the ICU staff, and above all, for ICNs and enrolled nurses who were used to structure and control. It worked well for some, while many ICNs had great difficulties with the deprioritisation and called in sick as a result. When ICMs clearly sanctioned deprioritisations, conflicts subsided.

The newly recruited staff were described by ICMs as experienced and skilled graduate nurses, but they did not comprehend the situation for the patients. They had had enough, as it was with their task and the care could not be as thorough as usual. ICMs also said that the ICU staff were tired and worn out from long-term heavy workloads, where holidays could not be granted and emotional outbursts and tears were common.

ICMs said that their ICU staff described how they felt that their work effort was subpar and that they did not have the opportunity to supervise and support newly introduced staff, something that caused them anxiety. Further along, ICU staff expressed feelings of being interchangeable checkers in a game, and pandemic-related sick leave and dismissal occurred in all ICUs. ICMs described the staff's reactions as signs of moral stress.

I think that is one of the reasons why there was quite a lot of sick leave among the nurses back then. That you are used to being able to give almost everything to the patients when it comes to the nursing part, the medical part. But here we had to make a concession due to the number of patients. ICM 11

The prioritisations led to health issues according to ICMs. During health checks, ICU staff were in need of psychological help and support. ICU staff were diagnosed with hypertension, diabetes, abnormal thyroid function, and abnormal weight gain. Difficulty sleeping or having an unreasonable need for sleep also took a heavy toll on ICMs, who described deteriorating health with memory problems, hypertension, diabetes, and prediabetes as an effect of critical care leadership during the pandemic.

But there were many people who, before I went on holiday, thought that I was sick. […] They have told me that now. The price has been too high. I feel as if I have aged five, ten years in these two years. ICM 12

## 6. Discussion

This study focuses on ICMs' experiences during the COVID-19 pandemic in several Swedish hospitals. The main findings indicate that intensive care experienced a period of challenges unlike any other in modern times. Specifically, the findings offer explanations of these experiences as constant change due to new guidelines, imbalance of patients and resources, and the pressure to persuade the ICU staff to continue working even when they could not see any improvement. The main findings were related to the heavy process of change that swept over the intensive care units and how that burden left behind both an individual and organisational fragility regarding human resources. The result is structured as a field of tension with the two themes: a dramatic change of the intensive care landscape and we could handle more than we thought, but at a steep price, and their respective subthemes (Figure 1). The subtheme getting ready to manage a new type of intensive care is expressed as adaptation to rapid changes needed in ICUs. ICMs stressed that initially it was a titillating experience, which offered something new and challenging where their problem-solving skills were put to the test. During crises, they found that adaptation must be developed continuously and diligently. In the findings, ICMs' roles in allocating resources were expressed clearly. Such resources included suitable premises, ICU staff with adequate competence, and obtaining equipment and drugs appropriate for the unknown during this intensive care crisis [21]. This has also been described in earlier studies [22–24], showing the strain on managers in critical care during the COVID-19 pandemic as the need for resources was shared by healthcare organisations internationally.

From the ICNs' point of view, this has been described, for instance, by Cadge et al. [8] as presenting the following themes: challenges of working with new coworkers and teams, challenges of maintaining existing working relationships, the role of nursing leadership in proving information and maintaining morale, and the importance of institutional-level acknowledgement of their work. These results show many similarities with the findings of the current study from the ICU managers' perspective. Results show that both the ICNs and ICMs were in a demanding new situation where the leadership and support were of great importance for all involved staff. In addition, the care environment and organisation in the ICUs have been highlighted in studies as being of importance in performing satisfying work during the COVID-19 pandemic [7, 24].

In the subtheme, the strain of introducing new staff as a challenge was depicted both for the ICMs as well as for the staff who were responsible for the close patient training. ICMs indicated that having colleagues who could not manage the work took its toll on the staff, mainly ICNs and intensive care assisting nurses. The challenges faced by ICMs overlapped with previous studies [25–27], but from the perspective of ICU staff. In Vera San Juan et al.' [27] study, the support from the work environment and more experienced colleagues appeared as one key principle for redeployed ICU staff. In Poortaghi et al.' [28] study, managers with a strong presence in the field were good role models for staff. According to Poortaghi et al.' [28] study, management of nursing staff during the COVID-19 pandemic included the appropriate recruitment, employment, replacement, and relocation of staff. To increase the quality of nursing care and patient safety, it is necessary to first examine the newcomers in terms of scientific and practical competencies and capabilities and then place them appropriately in combination with other staff in different departments [28].

White [24] showed the need for managers to focus on the well-being of the staff, especially in a means of reducing their anxiety and fears. The managers described ICU staff, especially ICNs, as having a need for constant communication about changing protocols for patient care and the need to reduce nurses' uncertainty about these changing interventions. Earlier studies [29, 30] also showed that managers' presence and availability were essential for their ability to support their staff. Managers who experienced high organisational stressors or high role stressors spent less time being present and available to their staff during the pandemic. In the current study, many ICMs could not be as present for staff as they desired due to their heavy administrative workload. As a consequence, this sometimes left ICMs feeling emotionally drained and wanting to be left alone. Considering the occurrence of burnout during the COVID-19 pandemic in ICNs and intensive care physicians [7, 31, 32], the feeling of wanting to be left alone expressed by ICMs' needs to be taken seriously by healthcare organisations or the ICMs may be the next group at risk for burnout.

The present study highlighted that supervising students learning to care for critically ill patients was an important, yet challenging issue. Consistent with previous studies that considered this issue [33], the present findings suggested ICMs need to balance demands for ICU staff supply during the pandemic and the risks for having students become infected due to lack of PPE. The present study underlined that allowing interns and postgraduate students to provide care during the COVID-19 offered them suitable patients who, in their severe illness, presented real-life challenges that are not always present in such quantity under such conditions and for such a long time.

Fredholm et al. [34] showed that postgraduate critical care nursing students learning in the ICU during the COVID-19 pandemic was a positive experience, as there were opportunities to care for many severely ill patients in need of mechanical ventilation, which allowed the students to connect to the patients and to experience authenticity in the ICU context. This shows that if students are given opportunities to form relationships in practice, even if the circumstances are not optimal, the general experience may work out positively.

Another crucial experience in supporting staff was to prioritise interventions, which ICMs had to face during the COVID-19. They had to support and impel ICU staff to deprioritise interventions such as oral care, pressure ulcer prevention, and change of dressing around intravenous access, which can be viewed as nonlife support interventions, yet very much connected to patient safety and high-quality care. Diminished patient safety and quality of care during the COVID-19 pandemic were described from an ICU staff perspective [35]. Patient safety risks and patient safety infringement were stressed, and these were Swedish ICU measures that had to be taken during COVID-19.

In the present study findings, the ICMs indicated that they took responsibility for certain actions in order to relieve the burden of moral distress from the shoulders of ICU staff in general and ICNs specifically. They also had to downplay their own worries as managers and stand up and give the appearance of security and strength in order to encourage staff to provide care that could be described as subpar. This result is in line with findings from other studies [23, 24], although managers in the former did not describe a striving for consensus as the ICMs did in the present study.

Nicola et al. [36] found strategies to curb COVID-19 were brought to fruition as a result of combining strong leadership and coordinated, intersectoral responses. These strategies included being prepared and acting quickly, testing, tracing, triage, and transparent communication. During COVID-19, leaders have adopted numerous best practice models and leadership strategies. Compassionate, open, and highly communicative leaders can strengthen people around them [36].

ICMs expressed that they had been part of an unparalleled healthcare crisis, yet they learned a great deal much during the pandemic. This is in line with Broome's observation [37] of nurse leaders revealing that although the pandemic created challenges and pressure beyond their comprehension, they made it through with new strength. Our study confirms the experience of nurses involved in the care of severely ill COVID-19 patients as previously reported. Silverman [38] underlined the difficulties expressed by ICMs as uncertainty of how to response to a new illness by which they were overwhelmed, fear of being infected, and the effects different policies and models had on nursing. The findings in other studies [7, 39, 40] further emphasised that ICNs also experienced insecurities, fears, worries, and moral distress due to the effects the pandemic had on critical care. Worth considering is what effect would the pandemic have had on a Swedish well-staffed intensive care unit with sufficient resources to receive more patients.

As depicted in this study, intensive care has experienced a period of challenges unlike any other in modern times. Findings show the effects of the dramatic changes brought about by the COVID-19 pandemic.

The pandemic is viewed as a crisis, and this term was also mentioned by the ICMs. Some of their actions and interventions can be viewed according to Burnison [41], who described leadership in a crisis with the purpose of accelerating through it. The crisis management actions in the current study are in line with the steps outlined by Kane et al. (2012) as *anticipate, navigate, communicate, listen, learn*, and *grow*. These were strategies whose *pearls and pitfalls* mirrored the actions taken by managers in another healthcare crisis prior to the COVID-19 pandemic (Kane et al., 2021). In the present study, those *pearls* could be described as the truly commendable actions in the themes. The pearls of *anticipate* and *navigate* can be described as the agile efforts made by ICMs to get ready for the unknown. The iterative upscaling of premises and resources would make it possible to remodel or build ICUs to manage the influx of patients. In *communicate* and *listen*, the pearls can be depicted as the clarity and honesty ICMs showed in the communication with the ICU staff, as they meant what they said but did not say everything they knew. They listened to the troubles reported by the ICU staff and strengthened the staff to provide care even when they were in doubt. Finally, *learn* and *grow* stand for the elevated insight acquired during the enormous pressure on the intensive care organisations during COVID-19. They managed it, even if the price was high.

### 6.1. Strengths and Limitations

The present study presents a sought after perspective regarding ICMs' experiences during the COVID-19 pandemic. Our study has managed to capture and describe the experiences of ICMs on different organisational levels, providing a varying and comprehensive assessment. The number of participants, given the total number of ICUs of the studied size in Sweden, is also a strength and contributes to trustworthiness and the transferability of findings. However, the interviews of the ICMs presented a challenge in regard to gaining access to in-depth personal experiences, as ICMs were eager to relate actual events more than their feelings emerging from these events. This was, however, detected early on during the interviews and allowed the two interviewers to respond by letting the ICMs first account for actual events, enabling more personal and deeper interviews later in the sessions. Such considerations contribute to the reflexivity in the study. The data analysis involved an open and lively discussion between authors, ultimately leading to consensus regarding findings. The latent two-sided structure of findings was a result of the independent analysis performed by the interviewing authors and, as such, considered a sign of both interpretative precision and rigor of findings.

## 7. Conclusion

The focus of this research was to draw attention to the heavy burden of managers responsible for ICUs during the COVID-19 pandemic. The pandemic constituted an extraordinary crisis in intensive care, characterised resulting from the expansive number of patients requiring advanced care. An extensive organisational change and its accompanying problems with prioritisation of resources have been depicted. Findings clearly show a field of tension between resources and demands, where premises and resources have been a rapid-changing entity of both material quality as well as in form of human resources. The prioritisation of resources has presented a heavy burden for the ICMs, who unreservedly all stated that they were able to handle more than they ever thought possible. However, the organisational, psychological, and physiological toll is evident. Research regarding the intensive care nurse shortages, workplace climate, sick leave, and intention to leave, moral distress, high workload, and burnout is suggested. Nevertheless, there were also examples of positive actions in the crisis situation. Further research illuminating the reasons for staff retention and successful crisis management is needed.

## Figures and Tables

**Table 1:** Characteristics of participating Swedish ICMs and workplace during COVID-19 pandemic.

Variables	*n* (%)
Sex	
Female	8 (67)
Male	4 (33)
Age in years	
40–50	6 (50)
51–60	2 (17)
61−	4 (33)
Spouse at home	12 (100%)
Children	11 (91%)
Experience as manager in years	
−5	6 (50)
6–10	5 (42)
15−	1 (8)
Profession	
Postgraduate nurse intensive care	9 (75)
Anesthesiologist	3 (25)
Participated in patient care themselves	
Yes	7 (58)
No	5 (42)
Intensive care accepted students	
Yes	8 (67)
No	4 (33)
ICU size during COVID-19, beds	
−10	4 (33)
11–20	5 (42)
21−	3 (25)

**Table 2:** Questions asked to ICMs (*n* = 12) about their experiences of care during the COVID-19 pandemic.

Can you please tell us about how reorganisations have affected your leadership during the pandemic?
How has the organisation handled supervision of students and new staff? What has that meant for you as a manager in terms of learning, introduction and continuing education?
Can you please tell us about the impact of the protective equipment on the work situation, learning and leadership?
Can you please tell us about priorities that have been made during the pandemic?
Can you please tell us about the support you have received as a manager?

## Data Availability

Access to data is restricted due to promises of confidentiality for participants.

## References

[1] Guttormson J. L., Calkins K., McAndrew N., Fitzgerald J., Losurdo H., Loonsfoot D. (2022). Critical care nurses’ experiences during the COVID-19 pandemic: a US national survey. *American Journal of Critical Care*.

[2] Nienhaus A., Hod R. (2020). COVID-19 among health workers in Germany and Malaysia. *International Journal of Environmental Research and Public Health*.

[3] World Health Organisation (2022). Emergencies. https://www.who.int/europe/emergencies/situations/covid-19.

[4] Oecd (2021). *European Observatory on Health Systems and Policies (2021), Sweden: Country Health Profile 2021, State of Health in the EU*.

[5] Nembhard I. M., Burns L. R., Shortell S. M. (2020). Responding to COVID-19: lessons from management research. *The New England Journal of Medicine Catalyst*.

[6] Marshall J. C., Bosco L., Adhikari N. K. (2017). What is an intensive care unit? A report of the task force of the World Federation of Societies of Intensive and Critical Care Medicine. *Journal of Critical Care*.

[7] Andersson M., Nordin A., Engstrom A. (2022). Critical care nurses’ experiences of working during the first phase of the COVID-19 pandemic–Applying the Person-centred Practice Framework. *Intensive and Critical Care Nursing*.

[8] Cadge W., Lewis M., Bandini J. (2021). Intensive care unit nurses living through COVID‐19: a qualitative study. *Journal of Nursing Management*.

[9] Carenzo L., Costantini E., Greco M. (2020). Hospital surge capacity in a tertiary emergency referral centre during the COVID-19 outbreak in Italy. *Anaesthesia*.

[10] Falk A. C., Nymark C., Göransson K. E., Von Vogelsang A. C. (2022). Missed nursing care in the critical care unit, before and during the COVID-19 pandemic: a comparative cross-sectional study. *Intensive and Critical Care Nursing*.

[11] Gab Allah A. R. (2021). Challenges facing nurse managers during and beyond COVID-19 pandemic in relation to perceived organizational support. *Nursing Forum*.

[12] Corona Commission (2021). Sub-report 2– Sweden during the pandemic. *SOU*.

[13] icuregswe (2023). Swedish intensive care registry. https://portal.icuregswe.org/siri/en/report/corona_inrapp.

[14] Hooker A. B., Etman A., Westra M., Van der Kam W. J. (2019). Aggregate analysis of sentinel events as a strategic tool in safety management can contribute to the improvement of healthcare safety. *International Journal for Quality in Health Care*.

[15] Waxman D., Chan E., Pillemer F., Smith T., Abir M., Nelson C. (2017). Assessing and improving hospital mass-casualty preparedness: a no-notice exercise. *Prehospital and Disaster Medicine*.

[16] Kim S. J. (2021). Crisis leadership: an evolutionary concept analysis. *Applied Nursing Research*.

[17] Graneheim U. H., Lundman B. (2004). Qualitative content analysis in nursing research: concepts, procedures and measures to achieve trustworthiness. *Nurse Education Today*.

[18] Tong A., Sainsbury P., Craig J. (2007). Consolidated criteria for reporting qualitative research (COREQ): a 32-item checklist for interviews and focus groups. *International Journal for Quality in Health Care*.

[19] Eurohealth (2020). COVID-19 health system response quarterly of the European observatory on health systems and policies. *Eurohealth*.

[20] Public Health Agency of Sweden (2020). *HSLF-FS 2020:12. Regulations and General Advice about Everyone’s Responsibility to Prevent the Spread of COVID-19*.

[21] Rubio O., Estella A., Cabre L. (2020). Ethical recommendations for a difficult decision-making in intensive care units due to the exceptional situation of crisis by the COVID-19 pandemic: a rapid review and consensus of experts. *Medicina Intensiva*.

[22] Ahmed F. R., Dias J. M., Al Yateem N., Subu M. A., Abu Ruz M. (2022). Lessons learned and recommendations from the COVID-19 pandemic: content analysis of semi-structured interviews with intensive care unit nurse managers in the United Arab Emirates. *Journal of Nursing Management*.

[23] Vázquez-Calatayud M., Regaira-Martínez E., Rumeu-Casares C., Paloma-Mora B., Esain A., Oroviogoicoechea C. (2022). Experiences of frontline nurse managers during the COVID-19: a qualitative study. *Journal of Nursing Management*.

[24] White J. H. (2021). A phenomenological study of nurse managers’ and assistant nurse managers’ experiences during the COVID-19 pandemic in the United States. *Journal of Nursing Management*.

[25] Christianson J., Guttormson J., McAndrew N. S., Calkins K. (2022). Impact of COVID-19 on intensive care unit nurse duty of care and professional roles: a qualitative content analysis. *SAGE Open Nursing*.

[26] Montgomery C. M., Humphreys S., McCulloch C., Docherty A. B., Sturdy S., Pattison N. (2021). Critical care work during COVID-19: a qualitative study of staff experiences in the UK. *BMJ Open*.

[27] Vera San Juan N., Clark S. E., Camilleri M. (2022). Training and redeployment of healthcare workers to intensive care units (ICUs) during the COVID-19 pandemic: a systematic review. *BMJ Open*.

[28] Poortaghi S., Shahmari M., Ghobadi A. (2021). Exploring nursing managers’ perceptions of nursing workforce management during the outbreak of COVID-19: a content analysis study. *BMC Nursing*.

[29] Gadolin C., Skyvell Nilsson M., Ros A., Törner M. (2021). Preconditions for nurses’ perceived organizational support in healthcare: a qualitative explorative study. *Journal of Health, Organisation and Management*.

[30] Gadolin C., Skyvell Nilsson M., Larsman P., Pousette A., Törner M. (2022). Managing health care under heavy stress: effects of the COVID-19 pandemic on care unit managers’ ability to support the nurses-A mixed-methods approach. *Journal of Nursing Management*.

[31] Stocchetti N., Segre G., Zanier E. R. (2021). Burnout in intensive care unit workers during the second wave of the COVID-19 pandemic: a single center cross-sectional Italian study. *International Journal of Environmental Research and Public Health*.

[32] Fumis R. R. L., Costa E. L. V., Dal’Col S. V. C., Azevedo L. C. P., Pastore Junior L. (2022). Burnout syndrome in intensive care physicians in time of the COVID-19: a cross-sectional study. *BMJ Open*.

[33] Ion R., Craswell A., Hughes L. (2021). International nurse education leaders’ experiences of responding to the COVID-19 pandemic: a qualitative study. *Journal of Advanced Nursing*.

[34] Fredholm A., Engströlm A., Andersson M., Nordin A., Persenius M. (2022). Learning in intensive care during the COVID-19 pandemic-postgraduate critical care nursing students’ experiences. *International Journal of Medical Education*.

[35] Bergman L., Falk A. C., Wolf A., Larsson I. M. (2021). Registered nurses’ experiences of working in the intensive care unit during the COVID-19 pandemic. *Nursing in Critical Care*.

[36] Nicola M., Sohrabi C., Mathew G. (2020). Health policy and leadership models during the COVID-19 pandemic: a review. *International Journal of Surgery*.

[37] Broome M. E. (2020). Leading through crisis. *Nursing Outlook*.

[38] Silverman H. J., Kheirbek R. E., Moscou-Jackson G., Day J. (2021). Moral distress in nurses caring for patients with COVID-19. *Nursing Ethics*.

[39] Andersson M., Fredholm A., Nordin A., Engstrom A. (2023). Moral distress, health and intention to leave: critical care nurses’ perceptions during COVID-19 pandemic. *SAGE Open Nursing*.

[40] Romero-García M., Delgado-Hito P., Gálvez-Herrer M. (2022). Moral distress, emotional impact and coping in intensive care unit staff during the outbreak of COVID-19. *Intensive and Critical Care Nursing*.

[41] Burnison G. (2021). *Leadership U: Accelerating Through the Crisis Curve*.

